# High quality permanent draft genome sequence of *Chryseobacterium bovis* DSM 19482^T^, isolated from raw cow milk

**DOI:** 10.1186/s40793-017-0242-6

**Published:** 2017-05-08

**Authors:** Sivan Laviad-Shitrit, Markus Göker, Marcel Huntemann, Alicia Clum, Manoj Pillay, Krishnaveni Palaniappan, Neha Varghese, Natalia Mikhailova, Dimitrios Stamatis, T. B. K. Reddy, Chris Daum, Nicole Shapiro, Victor Markowitz, Natalia Ivanova, Tanja Woyke, Hans-Peter Klenk, Nikos C. Kyrpides, Malka Halpern

**Affiliations:** 1 Department of Evolutionary and Environmental Biology, Faculty of Natural Sciences, University of Haifa, Haifa, Israel; 2 Leibniz Institute DSMZ—German Collection of Microorganisms and Cell Cultures, Braunschweig, Germany; 3 Department of Energy Joint Genome Institute, Walnut Creek, CA USA; 4 School of Biology, Newcastle University, Newcastle upon Tyne, UK; 5 Department of Biology and Environment, Faculty of Natural Sciences, University of Haifa, Oranim, Tivon, Israel

**Keywords:** *Flavobacteriaceae*, Psychrotolerant, Proteolysis, Lipolysis, Beta-carotene

## Abstract

*Chryseobacterium bovis* DSM 19482^T^ (Hantsis-Zacharov et al., Int J Syst Evol Microbiol 58:1024-1028, 2008) is a Gram-negative, rod shaped, non-motile, facultative anaerobe, chemoorganotroph bacterium. *C. bovis* is a member of the *Flavobacteriaceae*, a family within the phylum *Bacteroidetes*. It was isolated when psychrotolerant bacterial communities in raw milk and their proteolytic and lipolytic traits were studied. Here we describe the features of this organism, together with the draft genome sequence and annotation. The DNA G + C content is 38.19%. The chromosome length is 3,346,045 bp. It encodes 3236 proteins and 105 RNA genes. The *C. bovis* genome is part of the Genomic Encyclopedia of Type Strains, Phase I: the one thousand microbial genomes study.

## Introduction

*Chryseobacterium bovis* DSM 19482 ^T^ (=LMG 24227 ^T^; CIP 110170 ^T^), was isolated by Hantsis-Zacharov and Halpern [1] from raw cow milk when psychrotolerant bacterial communities in raw milk, and their proteolytic and lipolytic traits, were studied. This study revealed that 5% out of the culturable psychrotolerant bacterial communities belonged to the genus *Chryseobacterium*. *Chryseobacterium bovis* proliferates at low temperatures and produce heat-stable proteolytic and lipolytic enzymes which remain active after the milk pasteurization process. This may be a limiting factor in maintaining the flavor quality of fluid milk and its products [1]. Strain *C. bovis* H9^T^ DSM 19482 ^T^ was isolated in April 2004 from a modern farm equipped with automated milking facilities in northern Israel [2]. Three novel psychrotolerant *Chryseobacterium* species were isolated and identified from raw milk in the same study [1]: *C. bovis*, *C. haifense* and *C. oranimense* [2–4]. The genus *Chryseobacterium* [5] is a member of the family *Flavobacteriaceae* and currently consists of about 100 species with *Chryseobacterium gleum* as the type species. Species belonging to this genus exist in diverse environments such as milk, water, sludge, soil, animals, insects, plants and human samples [2, 6].

Here we describe a summary classification and a set of the features of the species *C. bovis*, together with the permanent draft genome sequence description and annotation of the type strain (DSM 19482 ^T^).

## Organism information

### Classification and features

**Fig. 1:**
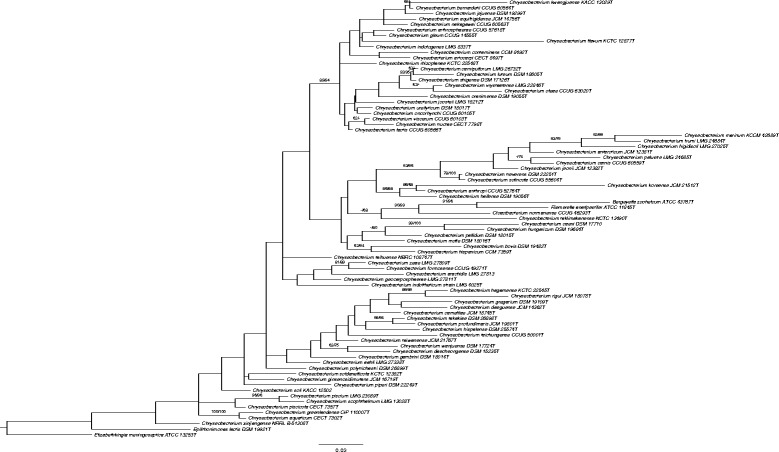
Phylogenetic tree highlighting the position of *Chryseobacterium bovis* relative to type species within the genus *Chryseobacterium*. Maximum likelihood (ML) tree inferred under the GTR + CAT model and rooted with *Elizabethkingia*. The branches are scaled in terms of the expected number of substitutions per site. The numbers above the branches are support values when larger than 60% from ML (left) and maximum parsimony (MP, right) bootstrapping. Phylogenies were inferred by the GGDC web server [34] available at (http://ggdc.dsmz.de) using the DSMZ phylogenomics pipeline [35] adapted to single genes. A multiple sequence alignment was created with MUSCLE [36]. ML and MP trees were inferred from the alignment with RAxML [37] and TNT [38], respectively. For ML, rapid bootstrapping in conjunction with the autoMRE bootstopping criterion [39] and subsequent search for the best tree was used; for MP, 1000 bootstrapping replicates were used in conjunction with tree-bisection-and-reconnection branch swapping and ten random sequence addition replicates

*C. bovis* strain DSM 19482 ^T^ shares typical characteristics of *Chryseobacterium* such as Gram-negative staining, occurrence as chemoheterotrophic rods and positive catalase and oxidase reactions. The strain contains flexirubin-type pigments, which are also typical for *Chryseobacterium* [2] (Table 1). The phylogenetic tree based on the 16S rRNA, also supports the fact that strain DSM 19482 ^T^ belongs to *Chryseobacterium genus* (Fig. 1).

**Table 1:** Classification and general features of *Chryseobacterium bovis* DSM 19482^T^ according to the MIGS recommendations [25], published by the Genome Standards Consortium [26] and the Names for Life database [27]

MIGS ID	Property	Term	Evidence code^a^
	Current classification	Domain *Bacteria*	TAS [28]
		Phylum *Bacteroidetes*	TAS [29]
		Class *Flavobacteriia*	TAS [30]
		Order *Flavobacteriales*	TAS [31]
		Family *Flavobacteriaceae*	TAS [32]
		Genus *Chryseobacterium*	TAS [5]
		Species *Chryseobacterium bovis*	TAS [2]
		Type strain DSM 19482^T^	TAS [2]
	Gram stain	Negative	TAS [2]
	Cell shape	Rod	TAS [2]
	Motility	Non-motile	TAS [2]
	Sporulation	Non-sporulating	IDS
	Temperature range	7–37 °C	TAS [2]
	Optimum Temperature	30–32 °C	TAS [2]
	pH range, Optimum	5.0–9.8; 6.5–8.5	NAS
	Carbon source	Glucose, lactose, Maltose	TAS [2]
MIGS-6	Habitat	Cow milk	TAS [2]
MIGS-6.3	Salinity, Optimum	0–2.5%; 0–1.75%	NAS
MIGS-22	Oxygen requirement	Facultative anaerobe	TAS [2]
MIGS-15	Biotic relationship	Unknown	TAS [2]
MIGS-14	Pathogenicity	Unknown	TAS [2]
MIGS-4	Geographic location	Northern Israel	TAS [2]
MIGS-5	Sample collection	2004	TAS [2]
MIGS-4.1	Latitude	32.635149	NAS
MIGS-4.2	Longitude	35.362050	NAS
MIGS-4.4	Altitude	Not reported	-

^a^Evidence codes - *IDA* Inferred from Direct Assay, *TAS* Traceable Author Statement (ie, a direct report exists in the literature), *NAS* Non-traceable Author Statement (ie, not directly observed for the living, isolated sample, but based on a generally accepted property for the species, or anecdotal evidence). Evidence codes are from the Gene Ontology project [33]

**Fig. 2:**
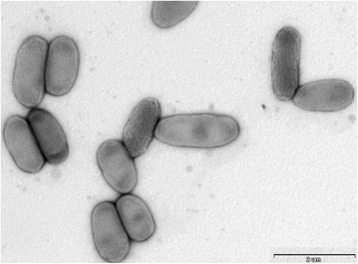
Electron micrograph of negatively stained cells of *Chryseobacterium bovis* strain DSM 19482^T^. Cells are nonflagellated rods. Bar, 2 μm

Cells of *C. bovis* strain DSM 19482 ^T^ are non-motile rods, measuring 0.5–0.9 μm in width and 1.1–2.3 μm in length (Fig. 2). After 48 h incubation on standard plate-count agar (SPC) at 30 °C in the dark, colonies are circular with entire edges, opaque, smooth and cream-colored. When light is provided during growth, colonies are yellow-colored because of the production of carotenoid-type pigments (absorbance peaks at 454 and 481 nm). They also contain small amounts of flexirubin-type pigments [2–4].

Growth is observed under anaerobic conditions on SPC agar containing 0.1% (w/v) potassium nitrate but not on SPC agar with the addition of 0.5% glucose (indicating that glucose is not fermented) [2]. The strain grows at 7–37 °C (optimum, 30–32 °C), with 0–2.5% NaCl (optimum, 0–1.75%) and at pH 5.0–9.8 (optimum, pH 6.5–8.5) (Table 1). *C. bovis* does not grow on MacConkey or cetrimide agar. Casein, aesculin and tributyrin are hydrolysed. Glucose, mannose, maltose, arabinose, mannitol, N-acetylglucosamine, gluconate and adipic and malic acids are assimilated. Acid is produced from D-glucose, maltose, D-lactose and D-mannose. Acetoin is produced; gelatin is hydrolyzed; H_2_S and indole are not produced; urea is not hydrolyzed; citrate is not utilized; and arginine dihydrolase, lysine and ornithine decarboxylases and tryptophan deaminase activities are absent. Alkaline and acid phosphatases, esterase (C4), esterase lipase (C8), leucine arylamidase, valine arylamidase, naphthol-AS-BI-phosphohydrolase, α-glucosidase, ß-galactosidase and cystine arylamidase activities are present [2].

#### Chemotaxonomic data

The major fatty acids of the type strains are: iso-C_15:0_; antesio-C_15:0_ and iso-C_17:0_ 3OH. Some strains in this species also possess iso-C_17:0_ ω9c as a major fatty acid [2].

## Genome sequencing information

### Genome project history

This organism was selected for sequencing based on its phylogenetic position [7] and is part of the study *Genomic Encyclopedia of Type Strains*, Phase I: the one thousand microbial genomes project [8]. The goal of the KMG-I study is to increase the coverage of sequenced reference microbial genomes [9]. The project is registered in the Genomes OnLine Database [10] and the permanent draft genome sequence is deposited in GenBank. Draft sequencing and assembly were performed at the DOE Joint Genome Institute (http://jgi.doe.gov/) using state of the art sequencing technology [11]. A summary of the project information is shown in Table 2.

**Table 2:** Genome sequencing project information

MIGS ID	Property	Term
MIGS 31.1	Finishing quality	Level 2: High-Quality Draft
MIGS-28	Libraries used	Illumina Std. shotgun library
MIGS 29	Sequencing platforms	Illumina HiSeq 2500, Illumina HiSeq 2500-1TB
MIGS 31.2	Fold coverage	230.5X
MIGS 30	Assemblers	Velvet (v. 1.2.07), ALLPATHS –LG (v. r46652)
MIGS 32	Gene calling method	Prodigal 2.5
	Locus Tag	LX71
	Genbank ID	FTPU01000000
	Genbank date of release	19-JAN-2017
	GOLD ID	Gp0103631
	BIOPROJECT	PRJNA262259
MIGS-13	Source Material Identifier	DSM 19482^T^
	Project relevance	GEBA-KMG, Tree of Life

### Growth conditions and genomic DNA preparation

A culture of DSM 19482 ^T^ was grown aerobically in DSMZ medium 381 [12] at 28 °C. Genomic DNA was isolated using a Jetflex Genomic DNA Purification Kit (GENOMED 600100) following the standard protocol provided by the manufacturer. DNA is available from the DSMZ through the DNA Bank Network [13].

### Genome sequencing and assembly

The draft genome was generated at the DOE Joint genome Institute (JGI) using the Illumina technology [14]. An Illumina std shotgun library was constructed and sequenced using the Illumina HiSeq 2000 platform which generated 7,888,518 reads totaling 1183.3 Mb. All general aspects of library construction and sequencing performed at the JGI can be found at (http://www.jgi.doe.gov). All raw Illumina sequence data was passed through DUK, a filtering program developed at JGI, which removes known Illumina sequencing and library preparation artifacts [15]. Following steps were then performed for assembly: (1) filtered Illumina reads were assembled using Velvet (version 1.2.07) [16], (2) 1–3 kb simulated paired end reads were created from Velvet contigs using wgsim (https://github.com/lh3/wgsim), (3) Illumina reads were assembled with simulated read pairs using Allpaths–LG (version r46652) [17]. Parameters for assembly steps were: (1) Velvet (velveth: 63 –shortPaired and velvetg: –very clean yes –exportFiltered yes –min contig lgth 500 –scaffolding no –cov cutoff 10) (2) wgsim (–e 0 –1 100 –2 100 –r 0 –R 0 –X 0) (3) Allpaths–LG (PrepareAllpathsInputs: PHRED 64 = 0 PLOIDY = 1 FRAG COVERAGE = 125 JUMP COVERAGE = 25 LONG JUMP COV = 50, RunAllpathsLG: THREADS = 8 RUN = std shredpairs TARGETS = standard VAPI WARN ONLY = True OVERWRITE = True). The final draft assembly contained 101 contigs in 96 scaffolds, totalling 3.3 Mb in size. The final assembly was based on 1152.3 Mb of Illumina data. 230.5X input read coverage was used for the final assembly.

### Genome annotation

Genes were identified using Prodigal [18], as part of the DOE-JGI genome annotation pipeline [19]. The predicted CDSs were translated and used to search the National Center for Biotechnology Information (NCBI) nonredundant database, UniProt, TIGRFam, Pfam, KEGG, COG and InterPro databases. The tRNAScanSE tool [20] was used to find tRNA genes, whereas ribosomal RNA genes were found by searches against models of the ribosomal RNA genes built from SILVA [21]. Other non–coding RNAs such as the RNA components of the protein secretion complex and the RNase P were identified by searching the genome for the corresponding Rfam profiles using INFERNAL [22]. Additional gene prediction analysis and manual functional annotation was performed within the Integrated Microbial Genomes (IMG) platform [23] developed by the Joint Genome Institute, Walnut Creek, CA, USA.

## Genome properties

The assembly of the draft genome sequence consists of 96 scaffolds amounting to 3,346,045 bp, and the G + C content is 38.19% (Table 3). Of the 3341 genes predicted, 3236 were protein-coding genes, and 105 RNAs. The majority of the protein-coding genes (69.95%) were assigned a putative function while the remaining ones were annotated as hypothetical proteins. The distribution of genes into COGs functional categories is presented in Table 4.

**Table 3:** Genome statistics

Attribute	Value	% of Total
Genome size (bp)	3,346,045	100.00
DNA coding (bp)	2,970,608	88.78
DNA G + C (bp)	1,277,778	38.19
DNA scaffolds	96	100.00
Total genes	3341	100.00
Protein coding genes	3236	96.86
RNA genes	105	3.14
Pseudo genes	0	0.00
Genes in internal clusters	2523	75.52
Genes with function prediction	2337	69.95
Genes assigned to COGs	1688	50.52
Genes with Pfam domains	2425	72.58
Genes with signal peptides	310	9.28
Genes with transmembrane helices	696	20.83
CRISPR repeats	2

**Table 4:** Number of genes associated with the general COG functional categories

Code	Value	% age	Description
J	167	9.12	Translation, ribosomal structure and biogenesis
A	0	0.00	RNA processing and modification
K	115	6.28	Transcription
L	110	6.01	Replication, recombination and repair
B	0	0.00	Chromatin structure and dynamics
D	24	1.31	Cell cycle control, cell division, chromosome partitioning
V	78	4.26	Defense mechanisms
T	56	3.06	Signal transduction mechanisms
M	189	10.32	Cell wall/membrane biogenesis
N	16	0.87	Cell motility
U	18	0.98	Intracellular trafficking, secretion and vesicular transport
O	88	4.81	Posttranslational modification, protein turnover, chaperones
C	103	5.63	Energy production and conversion
G	79	4.31	Carbohydrate transport and metabolism
E	147	8.03	Amino acid transport and metabolism
F	55	3.00	Nucleotide transport and metabolism
H	108	5.90	Coenzyme transport and metabolism
I	76	4.15	Lipid transport and metabolism
P	120	6.55	Inorganic ion transport and metabolism
Q	32	1.75	Secondary metabolites biosynthesis, transport and catabolism
R	148	8.08	General function prediction only
S	86	4.70	Function unknown
-	1653	49.49	Not in COGs

## Insights from the genome sequence

*C. bovis* DSM 19482 ^T^ showed the ability to hydrolyze casein and tributyrin [2] and these traits can also be observed in its genome. The following protease genes were detected: Membrane-associated serine protease, rhomboid family; ATP-dependent Clp protease ATP-binding subunit ClpB; Do/DeqQ family serine protease; ATP-dependent Clp protease ATP-binding subunit ClpX and transglutaminase-like enzyme, putative cysteine protease; ATP-dependent Lon protease (Lon functions in the cytosol) and cell division protease FtsH. The lipolytic properties of *C. bovis* DSM 19482 ^T^ are evident from the presence of the following genes: phospholipase/carboxylesterase; esterase/lipase superfamily enzyme and GDSL-like lipase/acylhydrolase.

*C. bovis* DSM 19482 ^T^ is producing carotenoid-type pigments under light conditions. Indeed, genes which are part of the carotenoid biosynthesis are found in its genome: phytoene desaturase (lycopene-forming), phytoene desaturase (neurosporene-forming), phytoene desaturase (zeta-carotene-forming), all-trans-zeta-carotene desaturase and beta-carotene 3-hydroxylase.

*C. bovis* DSM 19482 ^T^ was able to grow under anaerobic conditions when nitrate was provided. This ability is supported by the presence of the following genes: MFS transporter, NNP family, nitrate/nitrite transporter (two genes) and assimilatory nitrate reductase catalytic subunit.

Gliding motility properties are reflected by the presence of the genes that are exclusive to the *Bacteroidetes* phylum such as gliding motility-associated lipoprotein GldK and gliding motility-associated lipoprotein GldH. Another gene that supports the motility feature is the chemotaxis protein MotB gene.

Among the genes found in *C. bovis* DSM 19482 ^T^ genome are genes for resistance to different components. For example a gene for multidrug resistance protein, MATE family. Members of the Multi-Antimicrobial Extrusion (MATE) family function as drug/sodium antiporters. These proteins mediate resistance to a wide range of cationic dyes, fluroquinolones, aminoglycosides and other structurally diverse antibodies and drugs. These proteins are predicted to have twelve alpha-helical transmembrane regions. The Strain DSM 19482 ^T^ genome, also possesses a gene for cobalt-zinc-cadmium resistance protein CzcA. CzcA has a low cation-transport activity for cobalt and is essential for the expression of cobalt, zinc and cadmium resistance. Another gene found in the genome is a tellurite resistance protein TerC. TerC has been implicated in resistance to tellurium, and may be involved in efflux of tellurium ions. The quaternary ammonium compound-resistance protein SugE gene that is found in *C. bovis* DSM 19482 ^T^ genome encodes an efflux pump which confers resistance to cetylpyridinium, cetyldimethylethyl ammonium and cetrimide cations.

Resistance to antibiotics is revealed by the following genes: glycopeptide antibiotics resistance protein (plays a role in resistance to glycopeptide antibiotics such as vancomycin); MFS transporter, DHA1 family; tetracycline resistance protein gene; and Fusaric acid resistance protein-like gene, which is involved in the resistance (detoxification) of the fungal toxin Fusaric acid.

A gene for putative auto-transporter adhesin head GIN domain demonstrates the function of cell adhesion. Two genes indicate the possibility of *C. bovis* DSM 19482 ^T^ to produce a capsule, capsular exopolysaccharide family protein and polysaccharide export outer membrane protein.

## Conclusions

In the current study we characterized the genome of *C. bovis* strain DSM 19482 ^T^ that was isolated from raw cow milk [2]. *C. bovis* is a psychrotolerant bacterium which can grow at 7 °C, although its optimal growth temperature is higher (30–32 °C). After milk collection, the milk is kept in cold storage, and psychrotolerants dominate the bacterial flora. These bacteria possess extracellular enzymes, mainly proteases and lipases which contribute to the spoilage of dairy products, as their enzymes can resist pasteurization [1]. The *C. bovis* DSM 19482 ^T^ genome demonstrates that indeed, this genome encodes proteases and lipases which may play a role in milk products spoilage.

*C. bovis* strain DSM 19482 ^T^ produces a carotenoid pigment, a feature that was also observed for *C. haifense* [3], but not for other species in this genus. This trait could be used for the commercial production of carotene.

*C. bovis* DSM 19482 ^T^ genome demonstrated the strains' potential to produce a multidrug-resistance protein, resistance to cobalt, zinc, cadmium, tellurite, cetylpyridinium, cetyldimethylethyl ammonium and cetrimide cations as well as resistance to glycopeptide antibiotics, tetracycline and resistance to the fungal toxin fusaric acid. The whole-genome sequence of *C. oranimense* G311, a strain that was isolated from a cystic fibrosis patient, also demonstrated multi-drug resistance [24]. Indication for a capsule-forming ability was apparent in both *C. bovis* DSM 19482 ^T^ and *C. oranimense* G311. Sharma et al. [24] suggested that the resistance of *C. oranimense* G311 to colistin maybe due to the production of capsular polysaccharides.

## Abbreviations

GEBA: Genomic encyclopedia of Bacteria and Archaea
KMG: One thousand microbial genomes
MIGS: Minimum information about a genome sequence
NAS: Non-traceable
TAS: Traceable
